# Supplementary figures and images for: Dnmt1a is essential for gene body methylation and the regulation of the zygotic genome in a wasp
Source: PLoS Genet. 2022 May 6;18(5):e1010181. doi: 10.1371/journal.pgen.1010181 (PMC9075658; doi:10.1371/journal.pgen.1010181)

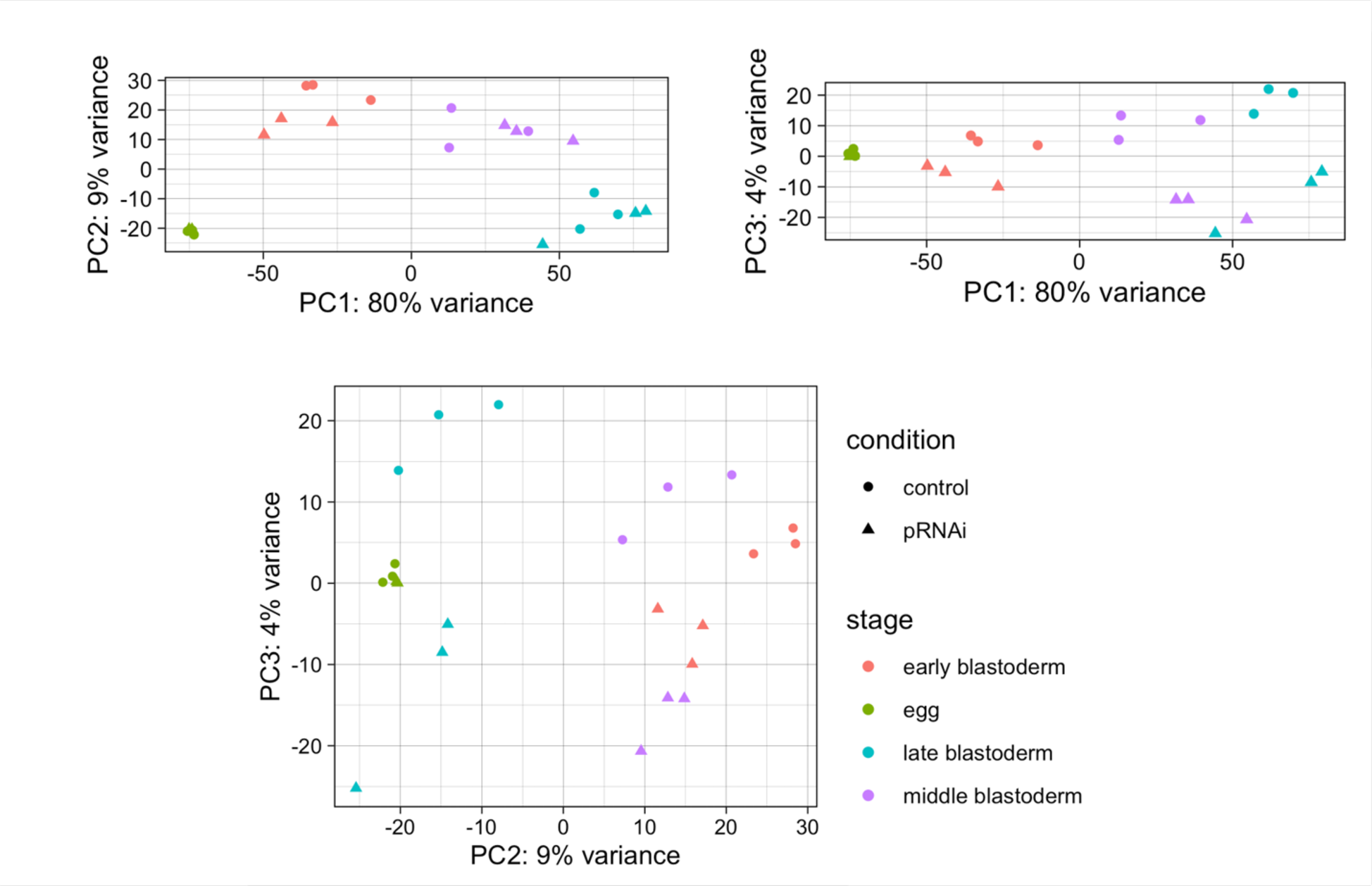

Supplement: S1 Fig — Circular points correspond to control samples while triangular points correspond to pRNAi samples. Green = eggs, salmon = early blastoderm embryos, purple = middle blastoderm embryos, blue = late blastoderm embryos. (TIF) [file pgen.1010181.s001.tif]

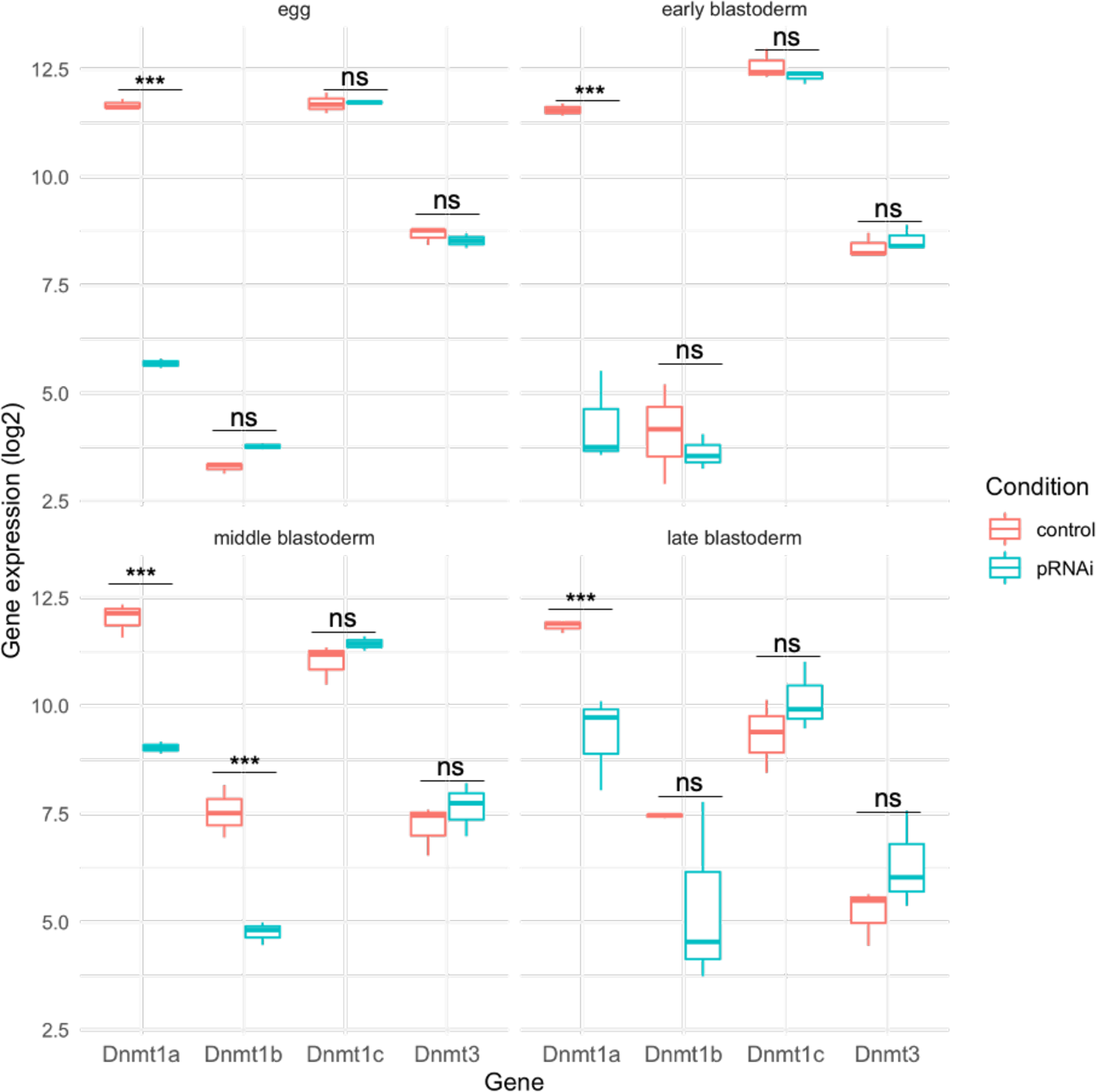

Supplement: S2 Fig — Control is labeled in salmon and pRNAi is labeled in teal. Nv-dnmt1a is significantly down-regulated in pRNAi samples in all stages. Nv-Dnmt1b, which depends on Nv-Dnmt1a for its gene body methylation, is significantly down-regulated in middle blastoderm embryos. Nv-Dnmt1c and Nv-Dnmt3 is not differentially expressed at any stage between pRNAi and control embryos. ns = not significant and triple asterisks (***) denote an FDR-adjusted p-value < 0.0001. (TIF) [file pgen.1010181.s002.tif]

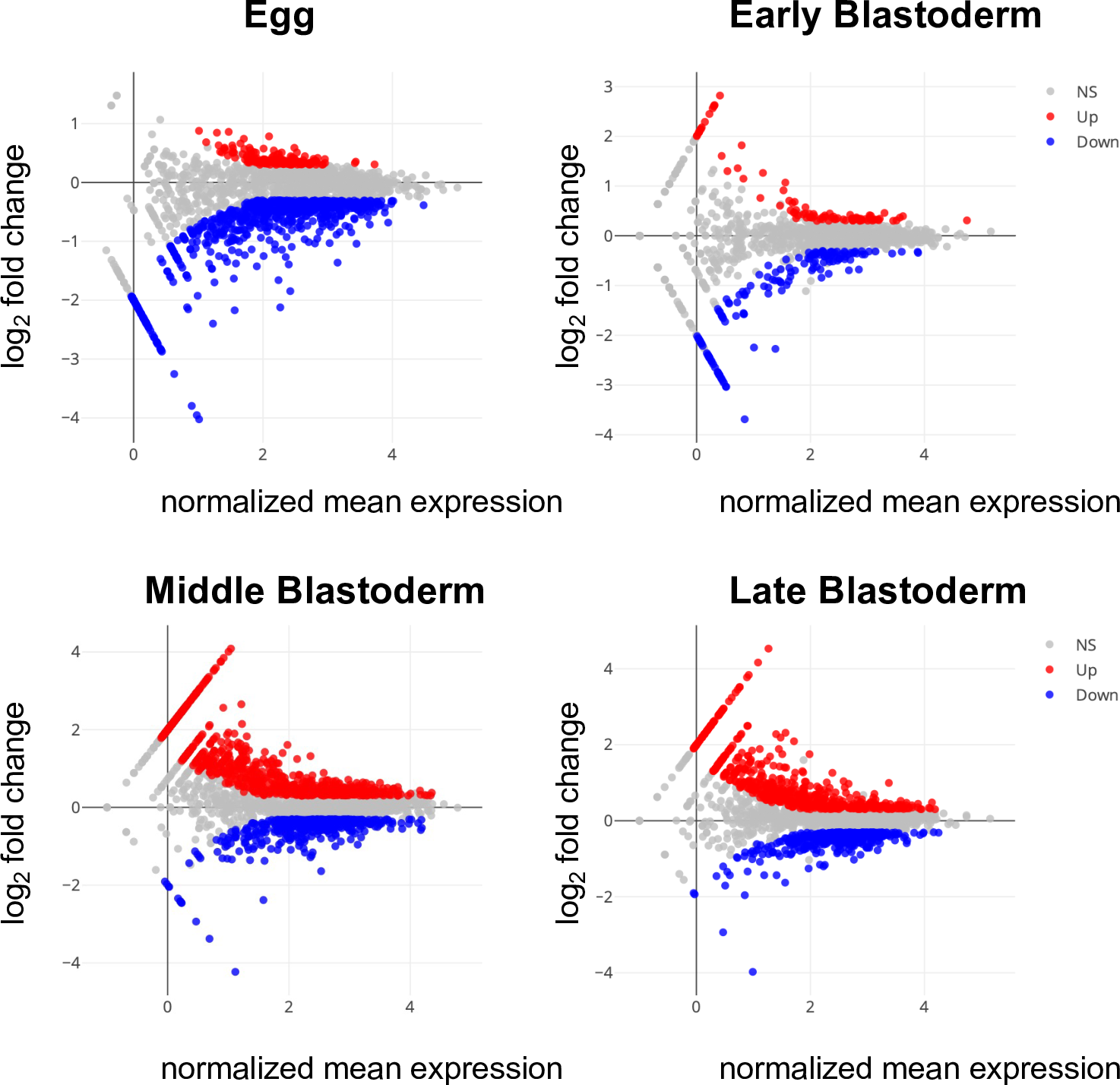

Supplement: S3 Fig — Red points indicate significantly up-regulated genes, while blue points indicate significantly down-regulated genes in pRNAi relative to control samples. Gray points are genes with no significant (NS) change in expression in pRNAi samples relative to control samples. (TIF) [file pgen.1010181.s003.tif]

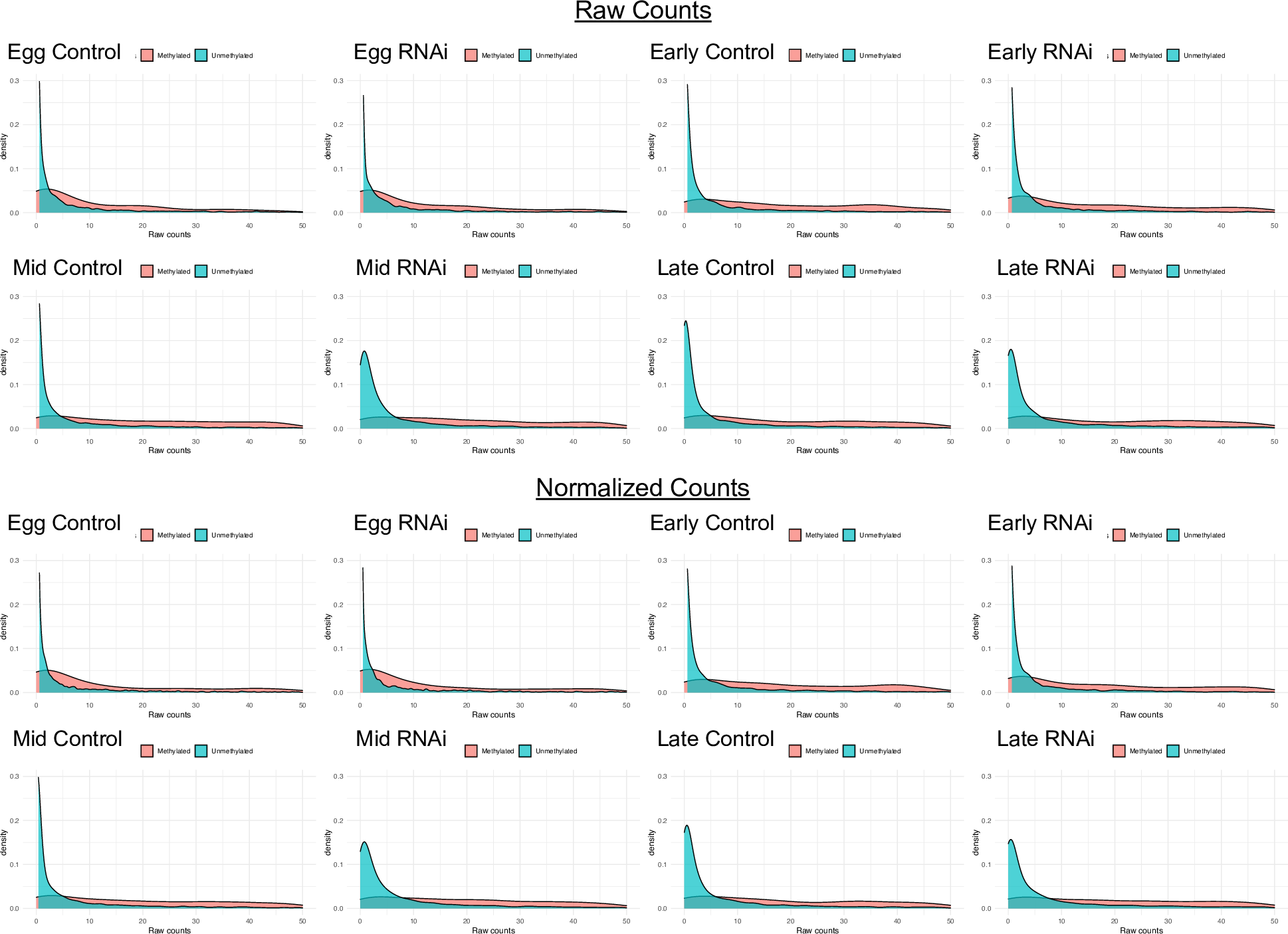

Supplement: S4 Fig — Density distributions of (A) raw transcriptome read counts and (B) normalized counts from DESeq2. Data are log2-transformed. Methylated (salmon) and unmethylated (teal) genes exhibit distinctive patterns of expression consistent with previous studies. The density distributions of the raw and normalized counts are highly similar. (TIF) [file pgen.1010181.s004.tif]

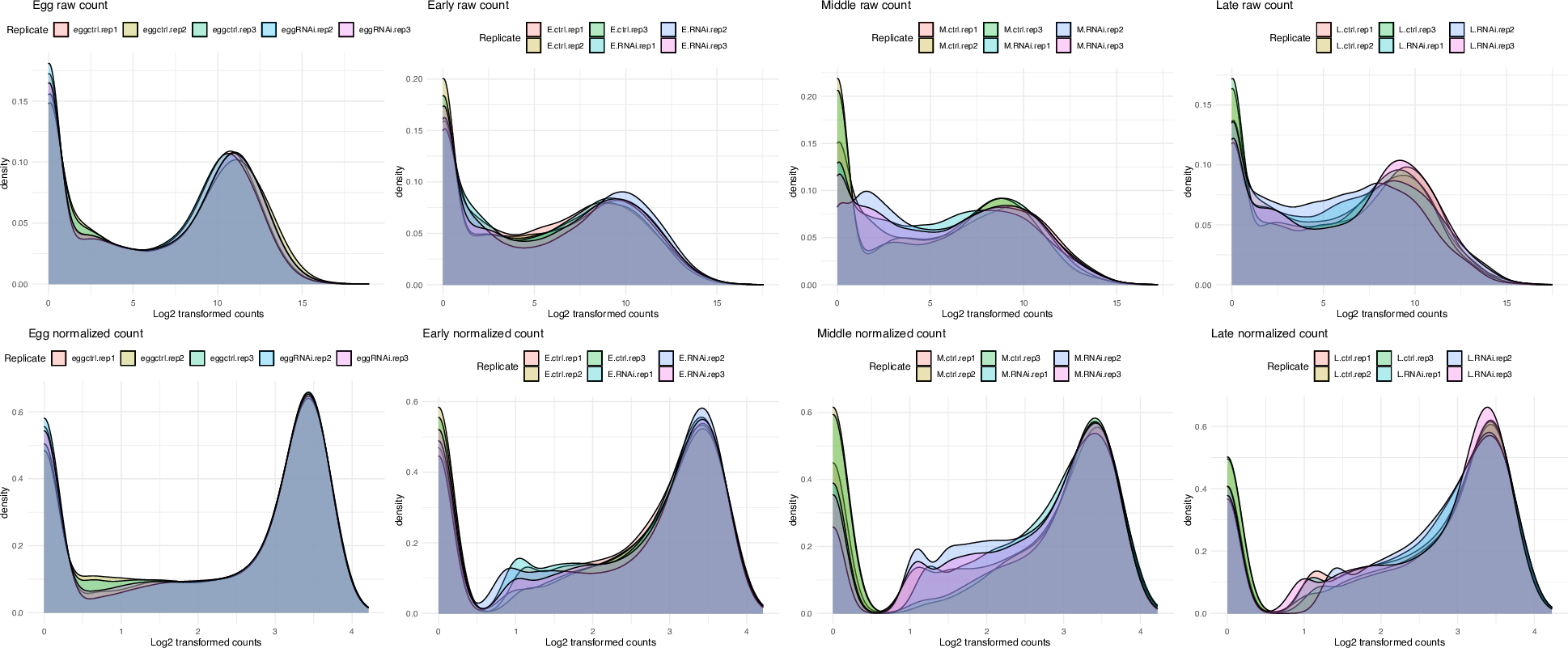

Supplement: S5 Fig — Density distributions of raw transcriptome read counts (top row) and normalized counts (bottom row) for all replicates and stages from DESeq2. Data are log2-transformed. (TIF) [file pgen.1010181.s005.tif]

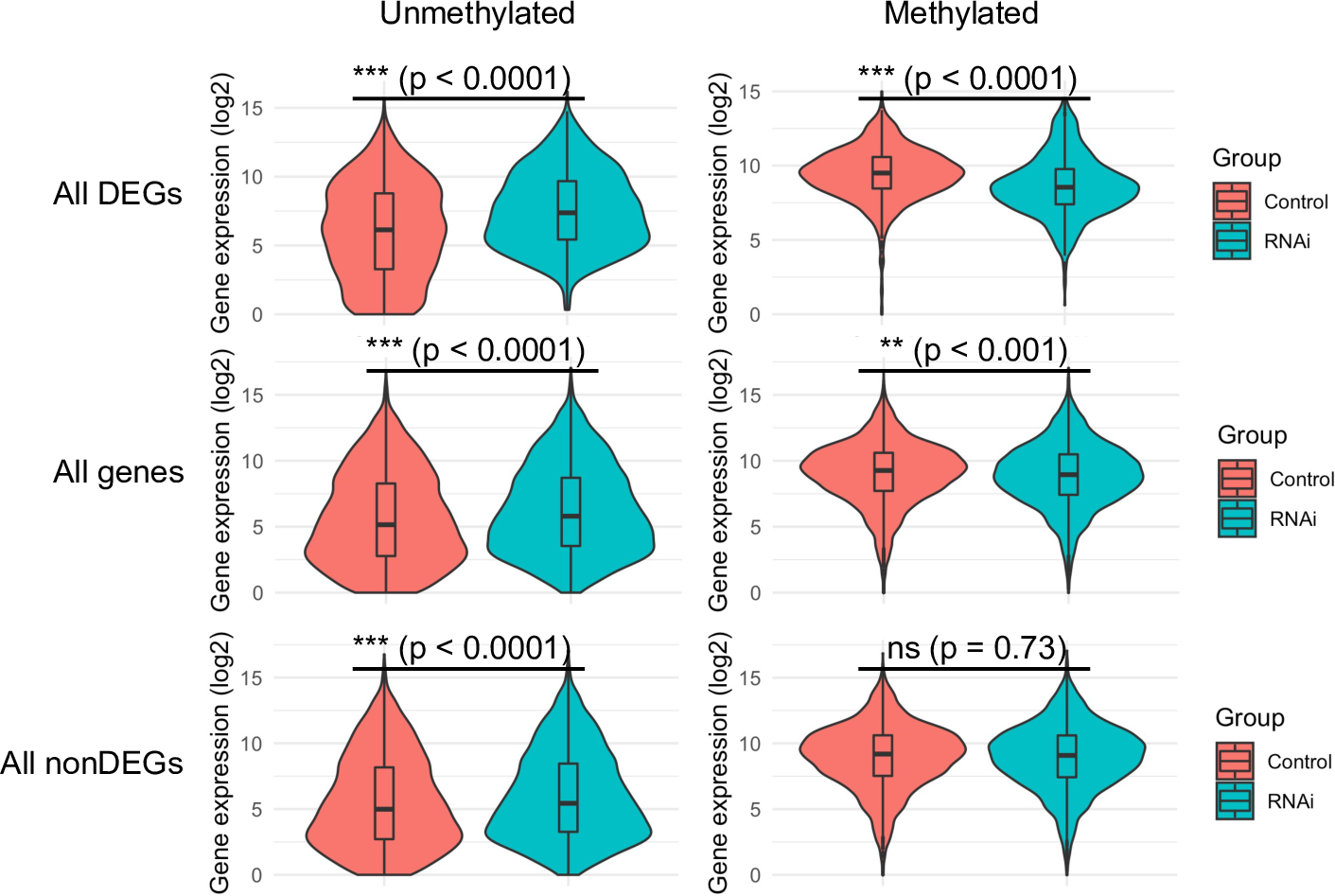

Supplement: S6 Fig — Top row, all differentially expressed genes (All DEGs), middle, all genes, and bottom, all non-differentially expressed genes (All nonDEGs). p values were determined by performing a 2-tailed T-test assuming unequal variance between RNAi and control. (TIF) [file pgen.1010181.s006.tif]

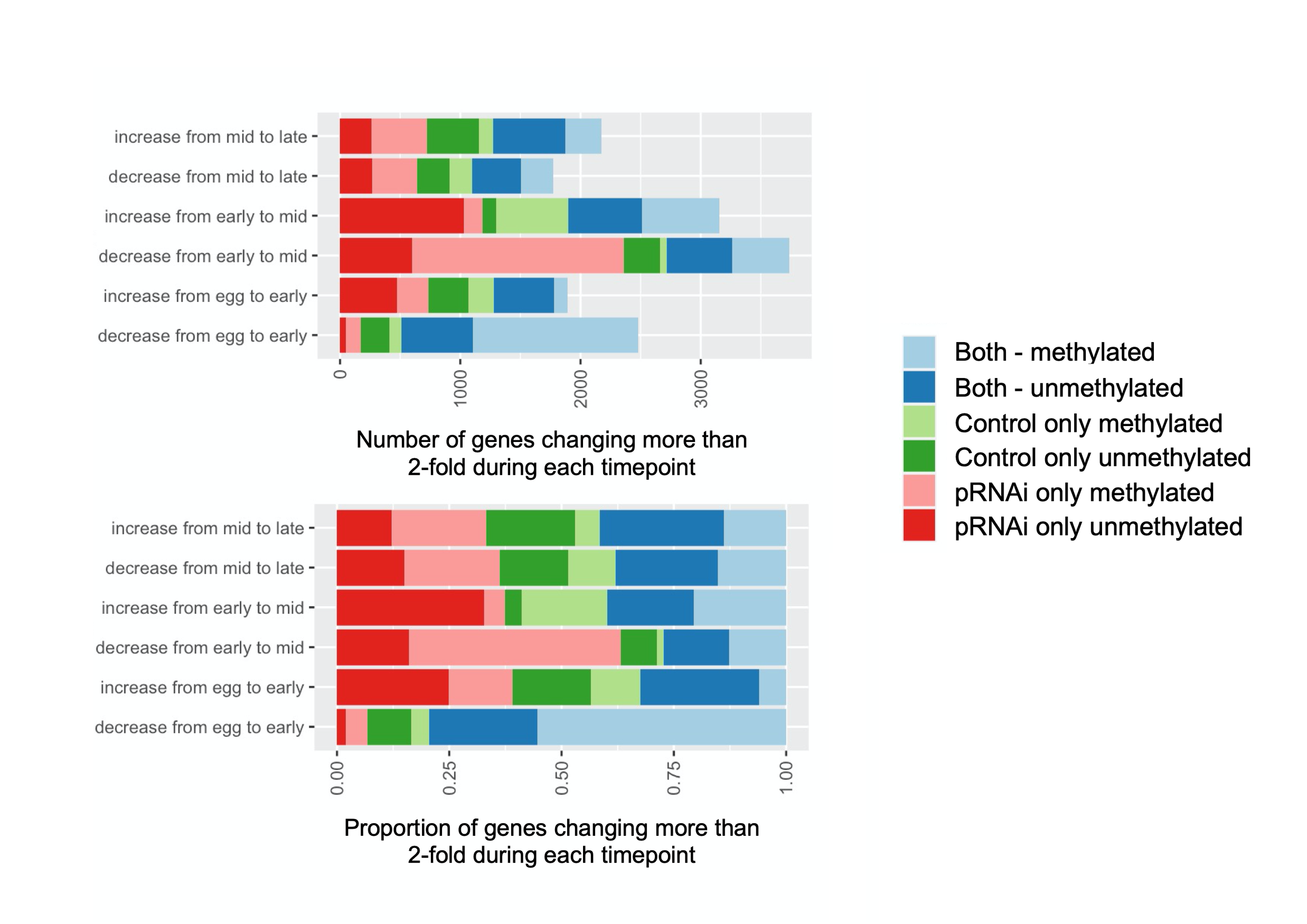

Supplement: S7 Fig — Number (top) and proportion (bottom) of methylated and unmethylated genes, in pRNAi samples, control samples or both, that increase or decrease 2-fold across each developmental transition. (TIF) [file pgen.1010181.s007.tif]

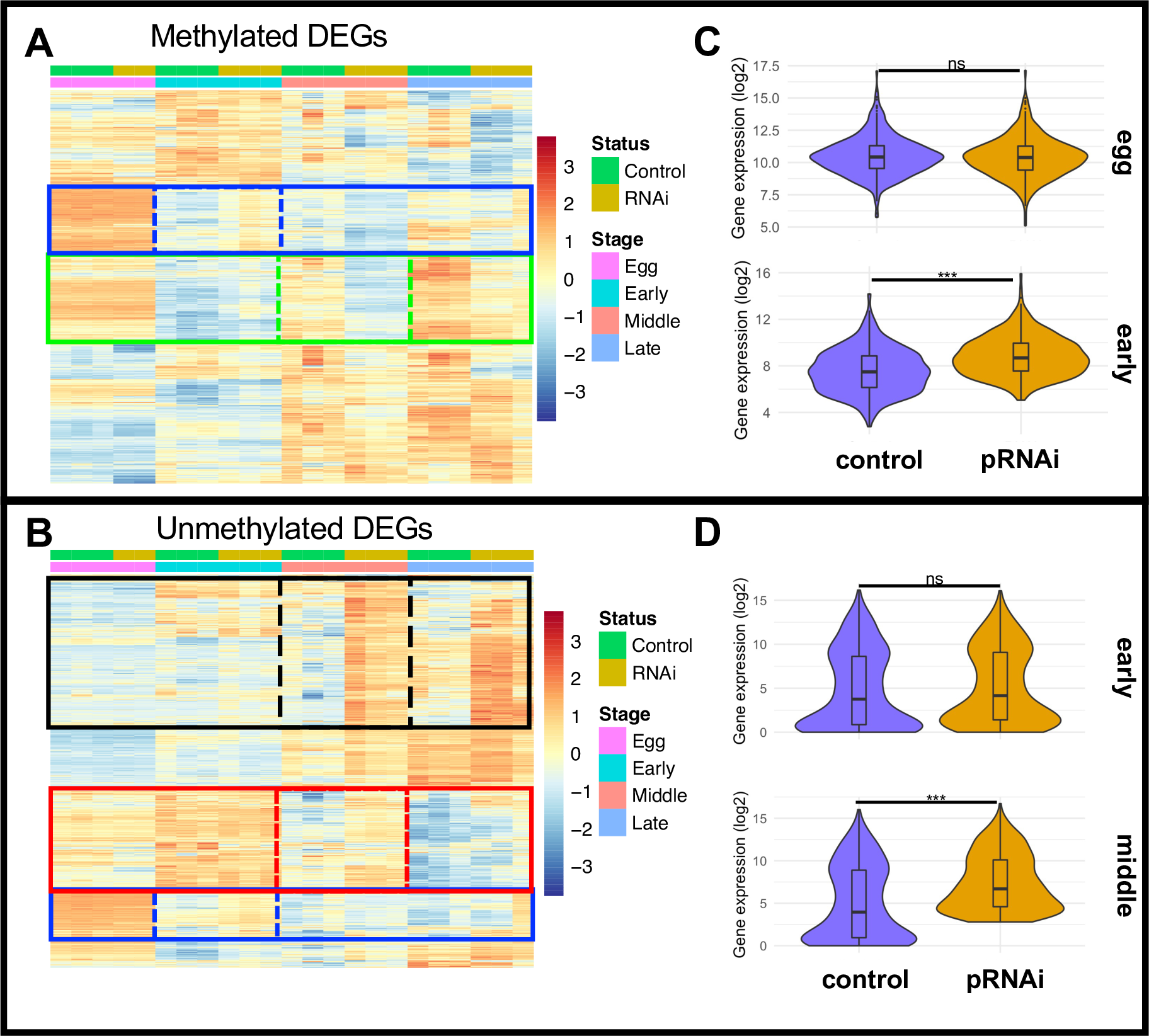

Supplement: S8 Fig — (A) Heatmap of 1769 hierarchically clustered methylated differentially expressed genes (mDEGs) and their expression over time in control and Nv-Dnmt1a pRNAi samples represented as Z-scores (warmer colors indicate higher than average expression, cooler, lower). Blue and green boxes on the heatmap highlight clusters of mDEGs that are of interest and discussed in the text. (B) Heatmap of 2135 hierarchically clustered unmethylated DEGs (uDEGs) and their expression over time in control and Nv-Dnmt1a pRNAi samples represented as Z-scores (as above). Black, blue, and red boxes on the heatmap highlight clusters of uDEGs that are of interest and discussed in the text. (C) Violin plots of 376 mDEGs in Nv-Dnmt1a pRNAi eggs and early blastoderm embryos. These mDEGs are significantly enriched in the early blastoderm Nv-Dnmt1a pRNAi samples relative to control early blastoderm embryos. Both comparisons were subjected to a t-test. ns = not significant (P>0.05), *** P<0.001 by t-test. (D) Violin plots of 213 uDEGs in control and Nv-Dnmt1a pRNAi early and middle blastoderm embryos, corresponding to the black box in (B). *** P<0.001 by t-test. (TIF) [file pgen.1010181.s008.tif]

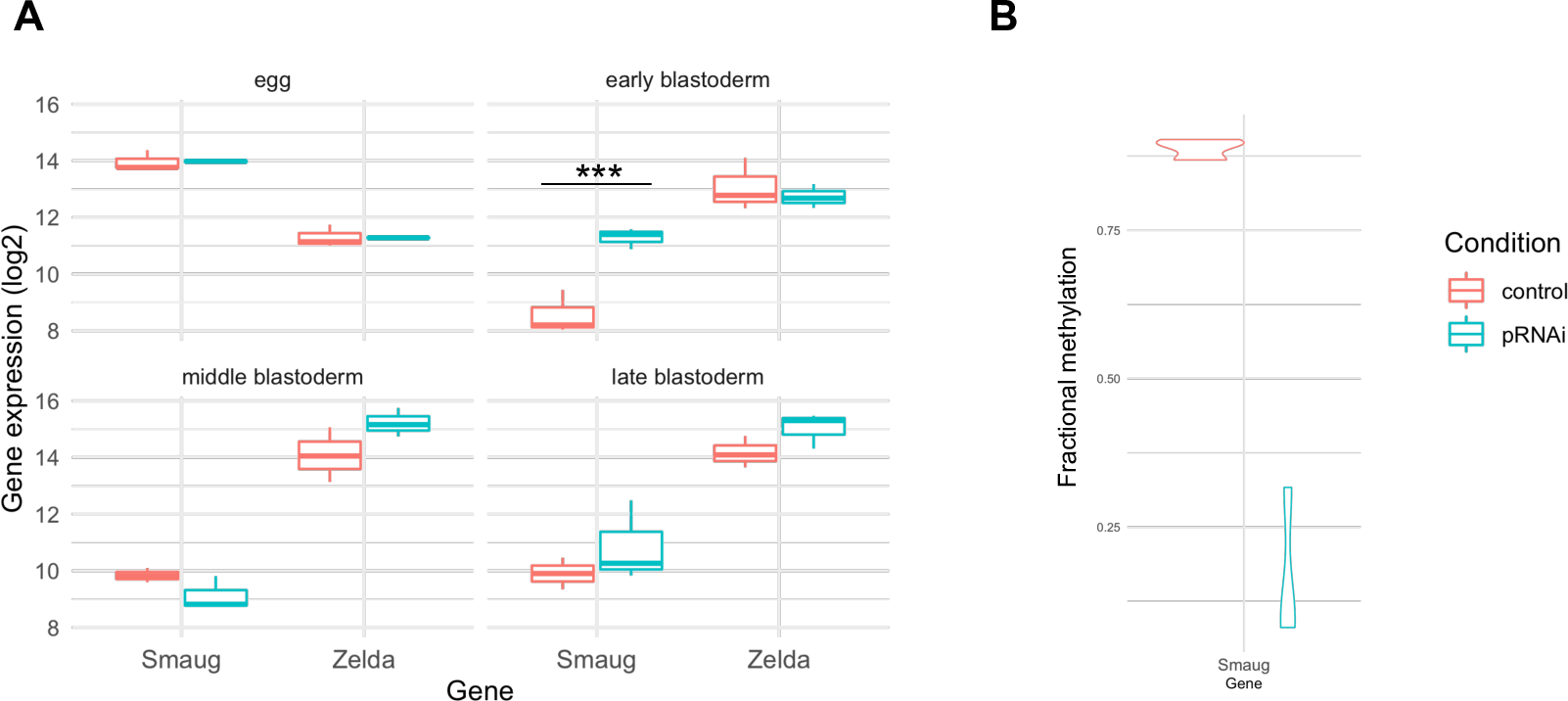

Supplement: S9 Fig — (A) Nv-smaug (LOC100118068) and Nv-zelda (LOC100118683) expression in egg, early blastoderm, middle blastoderm, and late blastoderm samples. Control is labeled in salmon and pRNAi is labeled in teal. Nv-smaug is only significantly up-regulated in the early blastoderm stage, while Nv-zelda is not differentially expressed at any stage. (B) Violin plot of Nv-smaug fractional methylation. Nv-zelda did not have any detectable levels of methylation in control or pRNAi samples and is considered an unmethylated gene (see Additional File 2). (TIF) [file pgen.1010181.s009.tif]

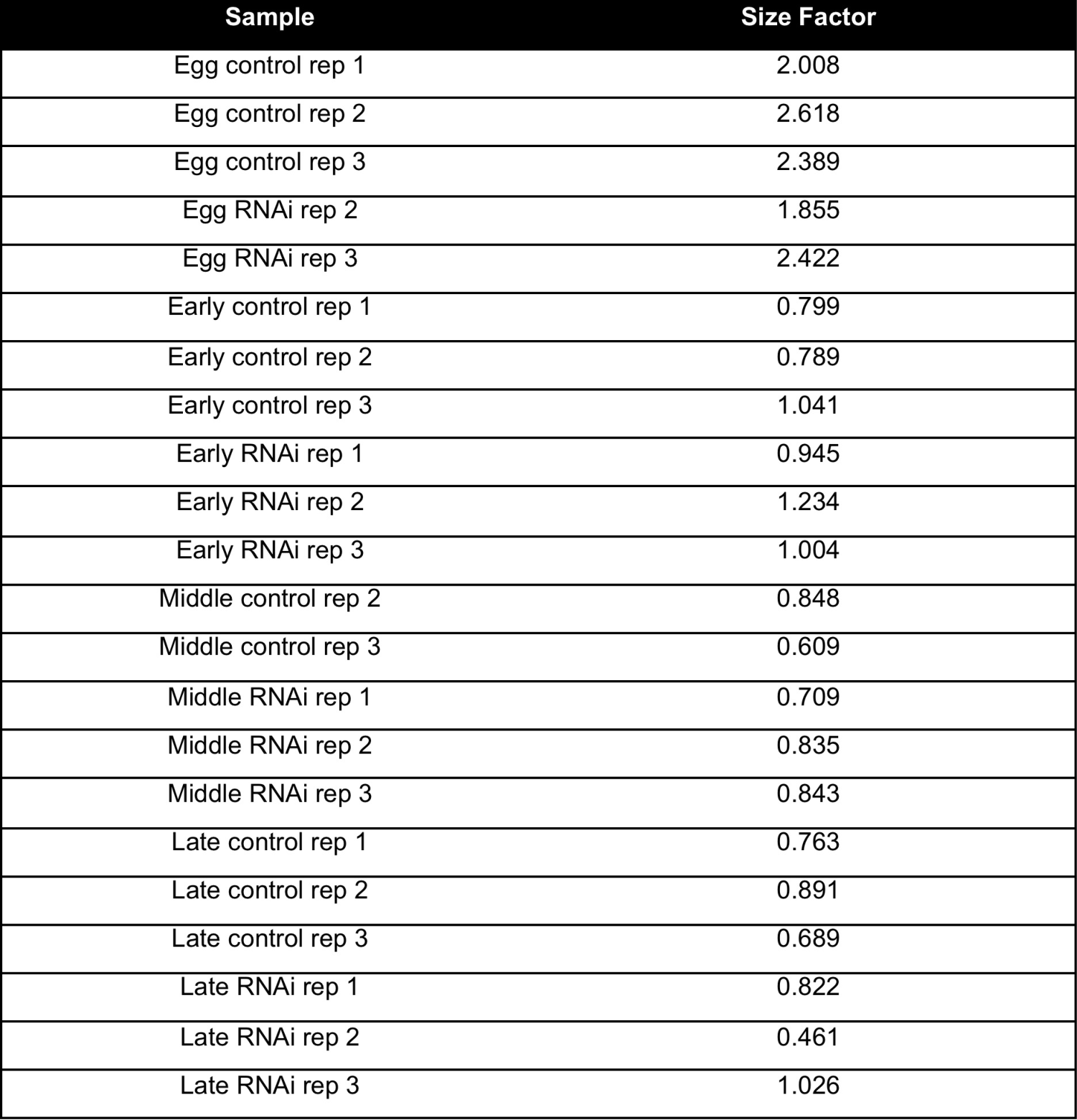

Supplement: S1 Table — (TIF) [file pgen.1010181.s010.tif]

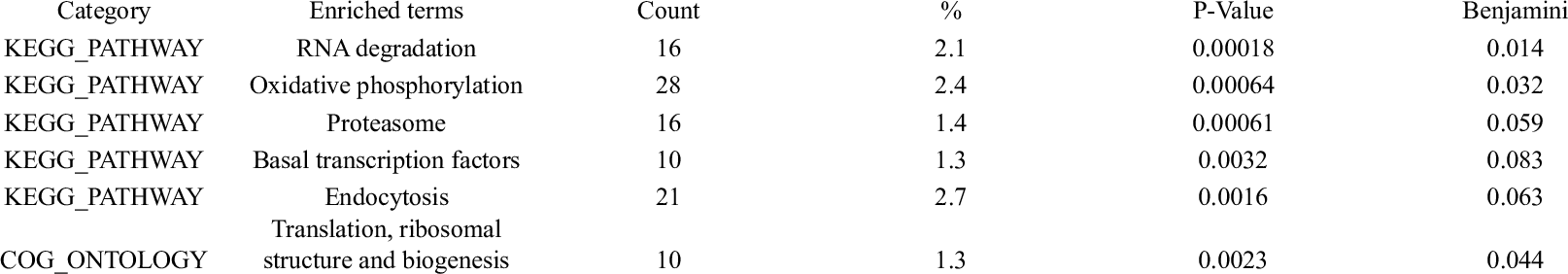

Supplement: S2 Table — (TIF) [file pgen.1010181.s011.tif]

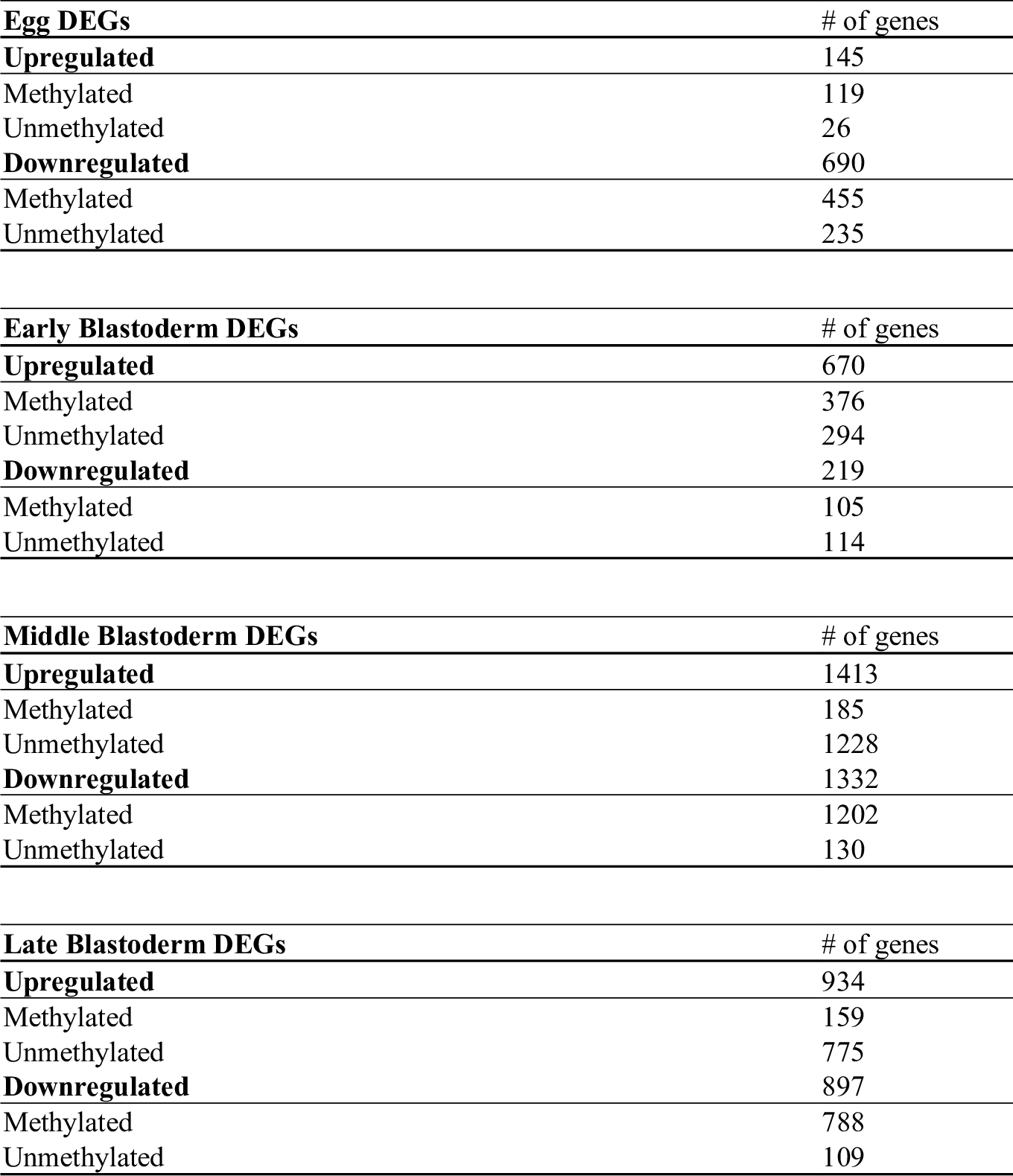

Supplement: S3 Table — (TIF) [file pgen.1010181.s012.tif]

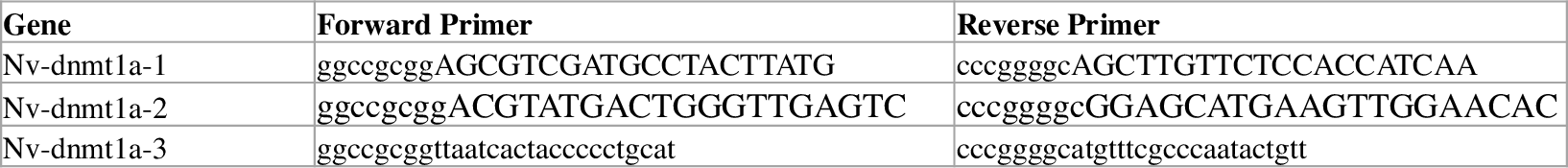

Supplement: S4 Table — (TIF) [file pgen.1010181.s013.tif]
