# Supplementary material for: Dnmt1a is essential for gene body methylation and the regulation of the zygotic genome in a wasp
Source: PLoS Genet. 2022 May 6;18(5):e1010181. doi: 10.1371/journal.pgen.1010181 (PMC9075658; doi:10.1371/journal.pgen.1010181)
Supplement: Attachment — Submitted filename: 120196_1_rebuttal_2273214_r6101x.docx [file pgen.1010181.s020.docx]

**Manuscript number:** RC-2021-01042R

**Corresponding author(s):** Jeremy Lynch

*[Please use this template only if the submitted manuscript should be considered by the affiliate journal as a full revision in response to the points raised by the reviewers.*

*If you wish to submit a preliminary revision with a revision plan, please use our "*[*Revision Plan*](https://docs.google.com/document/u/0/d/1HHp62Tfeb44nd3Gu_3ChB0b8gmMdEog7GjZcZFwHSiQ/edit)*" template.* ***It is important to use the appropriate template to clearly inform the editors of your intentions.****]*

## 1. General Statements [optional]

We have responded to the reviewers' thoughtful comments and criticisms and we have extensively revised the manuscript as a result. We feel this process has significantly improved the manuscript.

Point-by-point description of the revisions

**Response to Reviewers’ Comments**

We have substantially revised the manuscript following the suggestions by the reviewers. Below we provide detailed responses to reviewers’ comments. Our responses are shown in blue fonts.

Reviewer 1 Comments

However, the link with the maternal to zygotic transition is not that clear. I think some points would need further clarification:
Large scale changes in gene expression such as those described in this manuscript would benefit from rigorous normalization procedures, e.g. counting the amount of embryos used in each condition and using a matched number for each stage. Unclear if that has been done.

Response: The number of embryos collected at each stage between control and pRNAi samples were around 50. The same amount of total RNA was used for library preparation. The methods were updated to include this information. See lines 419-422.

In any case, the authors provide a couple of supplementary figures to address this, but they are a bit poor. Figure S1 has very low resolution, which makes it hard to read. Trying to compare raw count distributions using an x-axis of 5000 when most of the data is below ~200 is not fair, so that section is not very illustrative. As they nicely do in the other section, they log transform the values. But to visualize if some of the samples suffer from a bias in count distribution, they should show all replicates in the same stage (e.g. egg RNAi rep1, rep2, egg control rep 1, rep2) log transformed counts in the same plot, not dividing them into "methylated" vs "unmethylated genes" (y axis density - x axis log transformed raw counts). This should also be shown for the normalised values, to see if any possible shifts have been corrected with the default DEseq2 mode.

Response: We agree with the reviewer's comment, and we have produced the requested figure, making the data legible. See current Figure S4 also appended below.

***Figure S4****. Density distributions of (A) raw transcriptome read counts and (B) normalized counts from DESeq2. Data are log2-transformed. Methylated (salmon) and unmethylated (teal) genes exhibit distinctive patterns of expression consistent with previous studies. The density distributions of the raw and normalized counts are highly similar.*

I would request the authors to show how all replicates cluster in a PCA, to see how well they replicate and see if the developmental trajectory is preserved in the control samples. The size factors shown in Supplementary table show quite a lot of variation within stages/conditions, so some QC on the samples would be important.

Response: We updated the supplement and included 3-dimensional PCA for all control and pRNAi samples. The developmental trajectory of the samples separates clearly across PC1, while knockdown separates across PC2 and PC3 for the later stages. Please see the new Figure S1 appended below.

*Figure S1. 3-dimensional principal components analysis plot. Circular points correspond to control samples while triangular points correspond to pRNAi samples. Green = eggs, pink = early blastoderm embryos, black = middle blastoderm embryos, blue = late blastoderm embryos.*

Also, it would be nice to see some basic stats as total of million reads sequenced per replicate, and mappability, just to demonstrate that all these RNA-seq samples look "good" and comparable.

Response: Please see Additional File 7, in which we added alignment information, number of sequenced reads, and as well as additional details pertaining to our RNAseq experiments.

Another plot that would be a bit more informative than the volcano plots shown in Figure 4A is a MA-plot for each comparison. That would give a fair sense if the whole transcriptome is globally biased or just some genes are. The point here is that DEseq2 algorithm assumes that most genes are not differentially expressed to calculate the significance of changes, so finding a large set of genes that are not virtually affected by the treatment would be reassuring that there are no artifacts. This is not easily interpreted from Figure S2 (where unmethylated genes are always significantly biased). Finding so many "unmethylated genes" overexpressed could point out to a defective statistical test as the data violates the assumptions of the model, better to try to rule out that possibility.

Response: We updated the supplement to add MA-plots for each comparison. See plot below, which we included as Supplementary Figure 3. There are still many genes whose expression is not differentially expressed after pRNAi. We also information in Additional Files 1 and 3 containing all detected genes that weren’t significantly differentially expressed and their methylation status, showing that many unmethylated genes do not change in expression after pRNAi.

*Figure S3. MA plots visualizing gene expression in Nv-dnmt1a pRNAi and control samples in freshly laid eggs, early blastoderm embryos, middle blastoderm embryos, and late blastoderm embryos. Red points indicate significantly up-regulated genes, while blue points indicate significantly down-regulated genes in pRNAi relative to control samples. Gray points are genes with no significant (NS) change in expression in pRNAi samples relative to control samples.*

The developmental trajectory of the data should also be leveraged a bit better. Differential expression should be calculated for the various stages (egg to early blastoderm, early blastoderm to middle, etc) in control, and also in RNAi. That would first show clearly that the MZT is happening in control, and to what extent is disrupted by methylation loss/Dnmt1a knockdown. Genes could be clustered by expression dynamics (e.g. maternal, over-expressed in mid, over-expressed in late) which would render the impact on MZT a bit more precisely than the crude stage match comparisons.

Response: We have updated the manuscript to address these points. We added the figure below showing the clusters of methylated and unmethylated genes whose temporal expression patterns differ between control and pRNAi samples as Supplementary Figure 8. We also extensively updated the text describing these results (see lines 251 – 281).

***Figure S8****. Heatmaps and violin plots of differentially expressed genes in Nv-Dnmt1a pRNAi and control embryos. (A) Heatmap of 1769 hierarchically clustered methylated differentially expressed genes (mDEGs) and their expression over time in control and Nv-Dnmt1a pRNAi samples represented as Z-scores (warmer colors indicate higher than average expression, cooler, lower). Blue and green boxes on the heatmap highlight clusters of mDEGs that are of interest and discussed in the text. (B) Heatmap of 2135 hierarchically clustered unmethylated DEGs (uDEGs) and their expression over time in control and Nv-Dnmt1a pRNAi samples represented as Z-scores (as above). Black, blue, and red boxes on the heatmap highlight clusters of uDEGs that are of interest and discussed in the text. (C) Violin plots of 376 mDEGs in Nv-Dnmt1a pRNAi eggs and early blastoderm embryos. These mDEGs are significantly enriched in the early blastoderm Nv-Dnmt1a pRNAi samples relative to control early blastoderm embryos. Both comparisons were subjected to a t-test. ns = not significant (P>0.05), *** P<0.001 by t-test. (D) Violin plots of 213 uDEGs in control and Nv-Dnmt1a pRNAi early and middle blastoderm embryos, corresponding to the black box in (B). *** P<0.001 by t-test.*

Are these "housekeeping" genes affected by methylation really MZT? Or they were already present before? How many of the genes that really do get overexpressed in the mid/late blastoderm are affected by this RNAi?

Response: Wang et al., 2013 reported that methylated genes were significantly enriched for housekeeping functions, but they are not exclusively so. We have reconsidered using this term in the manuscript, since it is somewhat ambiguous. We found that roughly 95% of all methylated genes in the Nasonia genome were present among maternally deposited transcripts in the egg. Of the maternal transcripts that are down-regulated by the early blastoderm stage in our control samples, we see a large proportion of maternal transcripts that are upregulated in pRNAi early blastoderm samples relative to control, suggesting maternal transcript decay is partially affected (represented in Figure 4A and now shown in our new Supplementary Figure 8 appended above). Additionally, methylated genes represented almost all the down-regulated genes in our pRNAi samples in the middle blastoderm stage (during which ZGA is happening). Together, the improper decay of maternal transcripts and down-regulation of methylated genes during zygotic genome activation in our pRNAi samples indicates the maternal-to-zygotic transition is strongly affected. We updated our manuscript to include this point.

We also included a new Supplementary Figure 7 (appended below), where we show patterns in unmethylated and methylated genes whose expression changes more than 2-fold across developmental stages in control and pRNAi embryos. During the egg to early blastoderm transition, 2000 maternal transcripts are down-regulated, many of which are from methylated genes, which is expected and observed in both control and pRNAi embryos. However, by the early to middle blastoderm stage, we observed a decrease in 1758 methylated genes, which accounts for roughly 17% of the entire *Nasonia* genome and is seen only in our pRNAi embryos. This timepoint also corresponds with the amount largest transcriptional change, indicating it is within this time that the major wave of zygotic genome activation occurs—strongly suggesting *Nv-dnmt1a* positively regulates the expression of methylated genes during the MZT. Conversely, we see that *Nv-dnmt1a* has a likely indirect role in negatively regulating the expression of unmethylated genes, of which ~1000 show exclusive upregulation in pRNAi samples during the early to middle blastoderm transition. Both anomalies at the early-to-middle blastoderm transition are consistent with our general observations that mDEGs are mostly downregulated, while uDEGs are primarily upregulated.

*Figure S7. Number (top) and proportion (bottom) of methylated and unmethylated genes, in pRNAi samples, control samples or both, that increase or decrease 2-fold across each developmental transition.*

The authors propose: "This indicates that uDEGs that were effectively absent in control embryos were present at meaningful levels after Nv-Dnmt1a pRNAi. We propose that failure to either clear maternal RNA or repress transcription of these factors plays a crucial role in developmental failure at cellularization." Are these genes really maternal?

Response: This statement was meant as a summary of our observations of the egg to early blastoderm transition and later stage transitions. Some of the genes are maternal, but not necessarily the ones that increase from nothing to significant levels in the mid-blastoderm stage. We expanded on these points in lines 231 – 303.

Showing a global distribution makes it hard to interpret, so a bit more careful analysis would be necessary to show this (identifying the genes that go from non-expressed to expressed in the pRNAi).

Response: Figure S8 (appended above) also helps address this issue, and we added additional text to explain this statement better. We updated the text to clarify this point in lines 258-303

. The maternal genes referred to are those in the blue box in Figure S7A. All methylated genes in the early blastoderm stage that were up-regulated were initially present in the egg and had similar gene expression levels between control and pRNAi samples. Afterward, their levels fall in the control samples but do not fall to the same degree after pRNAi. For uDEGs, we observed two classes: 1) a set of genes that are zygotically activated in the early blastoderm stage in both pRNAi and control samples that are then cleared by the middle blastoderm stage in control samples but persist after pRNAi (red box). 2) we observe a class of uDEGs that activate in the middle blastoderm pRNAi samples (and persists into the late blastoderm pRNAi samples), while remaining at low levels in controls (black box).

3) The link between methylation and gastrulation is a bit dubious as the Dnmt1 knockdown in Tribolium castaneum, that lacks DNA methylation, also shows a similar early developmental phenotype as the one described in this manuscript (reference 53). Both species are somewhat derived (Tribolium lacks DNMT3, Nasonnia has several Dnmt1 paralogues), but the non-methylation roles of NvDnmt1a could be driving some of the phenotype in Nasonnia as well. I agree with the authors' discussion that the biased overlap between downregulated genes and methylated genes is quite striking (assuming the RNA-seq data is comparable, see point 1) and the correlation of methylation loss - silencing is also quite telling. Still, the connexion between methylation and transcription is not necessarily linked to the developmental phenotype, and this should be stated more clearly in the discussion. Irrespectively of what is the ultimate cause of the embryo arrest, the link with transcription is very interesting.

Response: We believe that our knockdown phenotype is distinct from the knockdown phenotype in *Tribolium castaneum.* As described by the authors of the Tribolium paper, the egg phenotype is a halting of development at the earliest cleavages, indicating a strongly maternal requirement for dnmt1a. Based on their description, most of the affected eggs are halted possibly even before the completion of meiosis, but at least before even an early blastoderm is formed. This is quite distinct from the very specific phenotype that we observe, which occurs only after the completion of all of the cleavage cycles known to be dependent on the maternal contribution of RNAs and proteins. We only see developmental defects once the very first processes dependent on the zygotic genome (cellularization and gastrulation) are trying to initiate. We updated the Discussion section to include these points (see lines 392 – 396).

4) Finding large number of differentially expressed genes in the egg is also a bit suspicious (the mother cells were already affected by treatment), as those transcripts are likely to have important effects on later development irrespectively of methylation. Discussing that a bit more carefully would be important.

Response: We clarified this point in our discussion and added Additional File 6 to show that these genes exist, but are small in number and magnitude of effect (see lines 354-356).

5) Are Smaug or Zelda methylated genes in Nasonia? How do they change in expression? (Smaug is mentioned, not Zelda). A genome browser shot of these genes would be good to see. Also, it would be nice to show a case of non-methylated genes that are upregulated, from Figure 5 that a substantial number of genes that had 0 expression in control become activated in RNAi. Showing examples of this would also indicate that this effect of global activation of unmethylated genes is more than artifact.

Response: Smaug is methylated and is upregulated during the early blastoderm stage, but is not differentially expressed at any other stage. Zelda is not methylated and was not differentially expressed at any stage. This information is supplied in Additional File 2 and 3. We now provide examples of genes that lose repression and become expressed after pRNAi in Additional file 4. These points are now mentioned in the revised manuscript (see lines 311 – 320).

6) Throughout the manuscript it is claimed that NvDnmt1a is responsible for GBM. Well, it is more likely that Dnmt1a is responsible for maintaining methylation on those genes, rather than the pattern itself. It is mostly a semantical distinction, but the consensus in the field is that Dnmt3 is what drives methylation to gene bodies, whereas Dnmt1 enzymes maintain CpG methylation wherever that is. That does not change the conclusions of this manuscript but should be stated clearly.

Response: We updated the conclusions to state this point. Also, showing expression of Dnmt1 paralogues and Dnmt3 in all the stages/conditions could be informative. We have done this, and the data is now included in Figure S2 (appended below).

*Figure S2. Expression levels of Nv-Dnmt1a (Dnmt1a), Nv-Dnmt1b (LOC100115455), Nv-Dnmt1c (LOC100123657) in egg, early blastoderm, middle blastoderm, and late blastoderm samples are shown, in comparison to those of Nv-Dnmt3 (LOC100114044) which is phylogenetically well separated from Dnmt1 in Nasonia and other insects (Li et al. 2018). Control is labeled in salmon and pRNAi is labeled in teal. Nv-dnmt1a is significantly down-regulated in pRNAi samples in all stages. Nv-Dnmt1b, which depends on Nv-Dnmt1a for its gene body methylation, is significantly down-regulated in middle blastoderm embryos. Nv-Dnmt1c and Nv-Dnmt3 is not differentially expressed at any stage between pRNAi and control embryos. ns = not significant and triple asterisks (***) denote an FDR-adjusted p-value < 0.0001.*

Some minor points:

1. Size factors of 2 are not "very small", a size factor of 2.5 implies quite a lot of variability (expected I guess when comparing the egg to the developing embryo). We agree and removed ‘very small’ from the text.
2. Line 217 There is an extra "." Fixed
3. Line 222 - "including the well l known MZT factors" -> the "I" is a typo. Fixed
4. Figure S1 legend - remove the line numbers found within the text. Fixed

5. Figure S2 legend - no mention of the statistical test used. Figure 5 then does not show the same type of statistical comparison. We updated Figure 5 to show significance and updated figure legends in both Figure S2 and Figure 5 describing the statistical test that we used.

6. Figure 4a - it would be nice to see a table with the numbers in each category (not immediately obvious from panel B and a bit hard to follow in the main text). Good suggestion, we added an additional table in our supplementary file.

7. MiddleBlastoderm-DEGs in the supplementary spreadsheet is empty and I think it should not (?). We fixed this error.

8. On the other supplementary spreadsheet, there are 6 columns named:

ctl1_methyl ctl2_methyl ctl3_methyl rnai1_methyl rnai2_methyl rnai3_methyl

That show plenty of NA values. What is that stage and why it has so many NA values? The NA values are shown for nonmethylated genes, and the stage is late blastoderm. We included an additional sheet with information to give more information about this file and edited the “methyl” column names to include the stage.

Reviewer 2 Comments

1. There are no quality assessments associated with the pRNAi experiment. I understand that this method was published before, nevertheless as a bare minimum it would be useful if the authors could produce some measure of knockdown efficiency. Those could include qPCR (or RNA-seq) assessments of Dnmt1a levels in the affected progeny, as well as levels of other Dnmts that could account for potential compensatory effects.

Response: See above response to Reviewer 1 who had a similar suggestion. A new supplementary figure has been added (Figure S2).

2. A rescue with wild type and catalytical mutant RNA would have been a very useful control that could directly test if the catalytic function of Dnmt is responsible for the observed phenotypes.

Response: We agree that this would be a great experiment, and as gene editing approaches improve in *Nasonia* such experiments will become more realistic. Currently however, such an experiment could not be conclusive with the current use of RNAi, where knockdown can still be variable and hard to quantify on an embryo by embryo basis (while also assaying for viability/methylation rescue). We would also need to find an mRNA that is both resistant to RNAi induced by our dsRNA, and yet can function the same as the native message. Significant optimization of embryonic injection would also be needed, since our experience with injecting mRNAs for fluorescent proteins did not consistently give ubiquitous expression, and often accumulate near the injection site. All these and probably more could be optimized more and more, but this could take the better part of a grad student career without a good guarantee of a clean result.

3. The authors need to show global measures of DNA methylation for all replicates to account for possible biological and technical variability.

Response: Methylation levels of all replicates are shown in Figure 3C and also in the Additional File 1 for each gene.

4. In figure 3, methylation data from only a single replicate is shown for most panels. It would have been much better to show average values from all replicates with confidence intervals (i.e in panel D). It is not clear what is shown in panel B. Is this whole genome DNA methylation or just GBs? If GBs, this should be stated. If not, these levels seem extremely high - much higher than a canonical non-vertebrate methylome. Again, it would be great to see these distributions for other replicates as well, even if as a supplemental figure.

Response: Only panel 3A shows a single replicate, the rest are derived from analyzing all replicates in either control or RNAi cases. For 3A, all control samples gave indistinguishable results. This has been clarified in the text and figure legend. Over 90% of methylated CpGs were found in gene bodies. This information is in Table 1, and we updated the text to point readers to table 1 for summary CpG statistics. Panel B represents the average fractional methylation of all CpGs in control samples and their corresponding positions in pRNAi samples. Panel D displays the pattern of mean fractional methylation in the first and last four exons and introns of all methylated genes in control and RNAi samples (which is divided into equal-sized bins). We re-worded the legend for Figure 3 to clarify these points.

5. The correlation in Figure 4C is weak (0.37). It is not clear if those are average methylation / transcription levels from the three replicates or if this is data from a single replicate only. If so, it would be interesting to see if this positive correlation can be observed across all replicates.

Response: The number represents the correlation between mean relative GBM change and mean relative expression change in all 3 pRNAi samples compared to all 3 control samples. We re-worded the figure legend for Figure 4 to clarify this point.

6. On page 7, when describing figure 5, the authors claim that RNAi resulted in a modest but significant shift of the distributions downward. It is not clear which statistical test was used to support that conclusion. While the authors only point out the major trends that fit with the hypothesis, it is worth noting that methylated DEGs in the early embryo do not follow the observed trend. i.e it appears that the knockdown slightly shifts the median upward.

Response: We updated Figure 5 and its legend to show the statistical test used for this analysis.

Reviewer #3 Comments

1. I think it will be helpful in the text and figure legend discussing figure 3 to discuss that the potentially methylated sites whose methylation fractions are being displayed in panels B and C are a portion of the sites considered in panel A, otherwise the numbers don't make sense.

Response: Panel A in Figure 3 only shows the bimodal distribution of fractional methylation sites in one control sample only, but this wasn’t clear in our legend. We updated both areas to clarify this point.
