# Supplementary material for: Dnmt1a is essential for gene body methylation and the regulation of the zygotic genome in a wasp
Source: PLoS Genet. 2022 May 6;18(5):e1010181. doi: 10.1371/journal.pgen.1010181 (PMC9075658; doi:10.1371/journal.pgen.1010181)
Supplement: Attachment — Submitted filename: ReviewerComments-03-20-22.docx [file pgen.1010181.s021.docx]

Addressing Reviewer Comments 03-18-22

**We thank the reviewers for their contributions to our manuscript. Our responses to requested changes are shown in blue font.**

Reviewer #1: The revised manuscript by Arsala et al., (Dnmt1a is essential for gene body methylation and the regulation of the zygotic genome in a wasp) presents interesting data regarding the role of gene body methylation (GBM) in gene regulation and development. In my view the most important and clear finding of this manuscript is “that after Nv-Dnmt1a pRNAi, the vast majority of methylated genes that are differentially expressed show reduced expression, and that the magnitude of reduced expression directly correlated with the degree of gene body methylation loss.” The authors also do an excellent job considering the impact of loss of GBM on aspects of development and discuss caveats to interpretation of impacts on development a of a broad disruption of gene regulation from their knockdown of DNA methyltransferase 1a (Nv-Dnmt1a). Another interesting aspect of this work is that, as the authors state, their work suggests that the role of GBM is different in different organisms indicating that over evolution the exact role of GBM can be different in different species. The revised manuscript is suitable for acceptance and will be of broad interest in the field.

Reviewer #2: In this revised version of the manuscript, the authors have addressed most of my points. Here I have some minor comments that might help:

1) The 3-D PCA in figure S1 is quite hard to interpret. Since it is in the Supplementary Material (so not much pressure on space), I would display PCA1 vs PCA2 in a 2D plot (and maybe another with PCA1 vs 3 or 2 vs 3 if required), which is way easier to interpret (as a general rule of thumb, 3D plots are quite confusing).

We have updated supplemental figure 1 to include 2-dimensional principal components analysis plots. Please see the new Supplementary Figure 1 appended below.

2) In Figure S7 – Please add a hyphen in the figure legend between “Both – methylated” or “pRNAi only – methylated”. I got confused trying to figure out how come some genes were “pRNAi only methylated”, wrongly interpreting it as those genes being only methylated in the pRNAi.

Updated the legend to make it more clear.

3) Some discussion on dnmt1b and dnmt1c could be interesting, these do not seem to be affected by the RNAi and are highly expressed during all the stages (and as previously shown, not required for development). Maybe those are more divergent sequences?

We have added a brief discussion of the evolution of the dnmt1 paralogs. None are significantly more diverged, and we simply propose that the paralogs have been subject to specialization and subfunctionalization

4) The links to the data are still not open.
We have updated the links to the data to make it public as of last week and confirmed it is now accessible. We are linking the DOI to the data as well. The data should now be accessible:
RNA - PRJNA701367
WGBS - PRJNA701143

Reviewer #3: The authors have successfully answered the majority of my queries (and those of other reviewers). In my opinion the manuscript can be accepted for publication in Plos Gen.
